# The remedial effect of soluble interleukin-1 receptor type II on endometriosis in the nude mouse model^☆^

**DOI:** 10.1016/S1674-8301(10)60007-3

**Published:** 2010-01

**Authors:** Liying Gao, Liang Sun, Yugui Cui, Zhen Hou, Li Gao, Jing Zhou, Yundong Mao, Suping Han, Jiayin Liu

**Affiliations:** a Center of Clinical Reproductive Medicine, the First Affiliated Hospital of Nanjing Medical University, Nanjing 210029; b Suzhou Municipal Hospital & Suzhou Medical Center for Maternal and Child Health, Suzhou 215002, China.

**Keywords:** Interleukin-1, soluble interleukin-1 receptor type II, endometriosis, nude mouse model

## Abstract

**Objective:**

Recent studies have shown that the local expression of soluble interleukin (IL) -1 receptor type II (sIL-1 RII) in endometrial tissue of women with endometriosis is decreased, and the depression of IL-1 RII was more significant in infertile women than that in fertile women with endometriosis. In this research, we investigated the remedial effect of sIL-1-RII administration on endometriosis in the nude mouse model.

**Methods:**

Nineteen nude model mice with endometriosis were randomly divided into three groups: group A was treated by intraperitoneal administration with only sIL-1 RII for two weeks, group B was similarly treated with only IL-1, and group C (control) was administered saline . After 2 weeks, the size of the ectopic endometrial lesions was calculated, and the expression of vascular endothelial growth factor (VEGF) and B-cell lymphoma leukemia-2 (Bcl-2) were detected by immunohistochemistry. The IL-8 and VEGF levels in the peritoneal fluid (PF) and serum were also measured by enzyme-linked immunosorbent assay (ELISA).

**Results:**

The mean size of ectopic endometrial lesion did not differ between the three groups (*P* > 0.05). Compared with the control, the expression of VEGF and Bcl-2 was significantly lower in group A, and higher in group B. In the three groups, the levels of IL-8 in the PF and serum were highest in group A, and lowest in group B.

**Conclusion:**

sIL-1 RII may suppresse hyperplasia of ectopic endometriosis, perhaps by reducing the expression of certain cytokines, such as VEGF, IL-8, and Bcl-2, which could provide a new clinical strategy for the treatment of endometriosis.

## INTRODUCTION

Endometriosis is a common gynecological disorder that affects 10%-15% of women of reproductive age and up to 50% of infertile patients. It is characterized by pelvic pain, dyspareunia and infertility as a result of the dystopic location of endometrial cells in tissues other than the uterine cavity[1]. The origin of endometriotic cells remains elusive and the most widely accepted theory on the aetiology of endometriosis is retrograde menstruation[2]. However, this phenomenon has also been described in healthy women and can be viewed as a physiological process[3],[4]. Thus factors other than retrograde menstruation are necessary for the development of endometriosis. Several lines of evidence indicate that the cytokines in the peritoneal fluid (PF) of women with endometriosis establishes a microenvironment for the development of the disease and undergoes a number of pathological changes, including inflammatory processes with locally and systemically altered functions of the immune system[5]–[7]. Increased levels of Interleukin (IL) -1ß mRNA expression have been identified in the peritoneal macrophages of patients with mild endometriosis[8]. IL-1 is a major proinflammatory cytokine which has been isolated from the PF of patients with endometriosis. It is mainly produced by monocytes and macrophages, and is believed to have an important role in endometriosis pathophysiology.

The IL-1 family consists of two distinct molecular forms, IL-1α and IL-1ß, which are encoded by different genes, but have comparable biological activities[9]. Cell activations in response to IL-1 are mediated via the IL-1 receptor type I (IL-1 RI), the functional signaling receptor[10]. However, IL-1 receptor type II (IL-1 RII) is not a signaling molecule and is reported to be a decoy target of IL-1. Moreover, IL-1 RII could be shed from the cell surface as a soluble molecule (sIL-1 RII) that captures IL-1 and inhibits IL-1 from binding to IL-1 RI, thereby neutralize the bioactivity of IL-1[11],[12],[13]. Recent studies have indicated that local expression of sIL–1 RII in endometrial tissue decreased in women suffering from endometriosis, and defective IL-1 RII was more significant in infertile women than in fertile women with endometriosis, which suggests its involvement in endometriosis associated infertility[14],[15].

The critical role of IL-1 in endometriosis has been confirmed in mice models, whereby successful implantation is blocked by administration of exogenous IL-1 receptor antagonist (IL-1ra), another natural specific inhibitor for IL-1 signaling[16]. In endometriotic stromal cells, IL-1β has been shown to stimulate angiogenic factors such as vascular endothelial growth factor (VEGF) and IL-8, cytokines endowed with neutrophil chemotactic and angiogenic properties[17], which is not observed in normal endometrial stromal cells. Our previous study also revealed that IL-1β significantly increased the production of IL-6 and IL-8, and this effect could be counteracted by recombinant adenovirus IL-1 RII (rAd-RII)[18]. Furthermore, we showed that overexpression of rAd-RII could suppress Bcl-2 expression in ectopic endometrium, suggesting that IL-1 RII may promote apoptosis of endometriotic stromal cells through inhibition of B-cell lymphoma leukemia-2 (Bcl-2) expression. The Bcl-2 gene was defined as a new class of proto-oncogenes that block cell death in the absence of cell proliferation. Studies have shown that increased expression of the Bcl-2 protein is present in the proliferative eutopic endometrium of patients with endometriosis[19].

The purpose of this study was to evaluate the therapeutic potential of sIL-1 RII in counteracting the effect of IL-1 using an *in vivo* nude mouse model of experimentally-induced endometriosis.

## MATERIALS AND METHODS

### Acquisition of human tissues

Eutopic endometrial tissues at the proliferative phase of the menstrual cycle were obtained from 5 women (aged from 23 to 35 years) undergoing laparoscopy for endometriosis. Endometriosis in these 5 women was staged as II or III during the laparoscopy, according to the revised American Fertility Society classification system[20]. All participants had regular menstrual cycles, had not received hormonal treatment, and had not been pregnant, breast-feeding, or using an intrauterine device for the past 3 months. Endometrial biopsies were placed immediately into a culture medium and transferred to the laboratory. Written informed consent was obtained from each patient using consent forms and protocols approved by the Ethics Committee of the First Affiliated Hospital of the Nanjing Medical University.

### Transplantation of the endometrial fragments into nude mice

Nineteen 6- 8-week-old female nude mice purchased from the Shanghai Laboratory Animal Corporation were maintained in a barrier unit in a well-controlled pathogen-free environment with regulated cycles of light/dark (12 h/12 h). All equipment and food entering the barrier was autoclaved. Mice had free access to food and water. Approval from the Animal Ethics Committee of the Nanjing Medical University was obtained before the study.

Mice were weighed, and anesthetized with intraperitoneal injections of pentobarbital. The endometrial tissue fragments were washed three times with PBS and cut into 2-3 mm^2^ sections that were implanted with a suture of polypropylene 3/0, in the parietal peritoneum in two sections on each side of the midline incision with a separation of 0.5-1.0 cm. The abdominal wall was then closed with Prolene 3-0 non-absorbable polypropylene sutures (Holycon, Nantong, Jiangsu, China). Each mouse received an intramuscular injection of benzestrofol (2 mg/Kg) every 4 days from the day of tissue transplantation for 2 weeks. All animal studies were approved and conducted in accordance with Institutional Animal Care Committee guidelines.

### Drug administration

After transplantation with endometrial tissue, nineteen nude mice were randomly divided into three groups: group A (*n* = 7) was treated with only sIL-1 RII for two weeks, group B(*n* = 6) was treated with only IL-1 for the same time period and group C(*n* = 6) was treated with saline as control. The optimal dosage was unknown due to the lack of previous studies on sIL-1 RII for experimental endometriosis. Therefore, we based the dosage on sIL-1 RII treatment in the experimental orthodontic tooth movement in rats, namely 2 µg/215 g[21]. Each group of mice was treated with an intraperitoneal injection of 0.2 ml of their respective treatments every other day for 2 weeks. Treatment with human recombinant IL-1 (5 ng/kg) (R&D System, USA) and human recombinant sIL-1 RII (25 µg/kg) (R&D System) was started at day 1 after tissue transplantation. Mice in the control group received the same volume of saline.

### Evaluation of ectopic uterine tissue

Two weeks after the beginning of treatment, animals were sacrificed and the lesions were collected to determine the growth of each implant based on the area of lesions. The growth of lesions per mouse was measured using the average size of the four implants, which were calculated by measuring two perpendicular diameters (a×b, length and width) with a caliper and the area of each lesion was calculated by the formula of an ellipse (a×b×II/4)[22]. The person performing the laparotomy and the evaluation of the endometriotic implants had no prior knowledge of the animal group allocation.

After measuring, the lesions were immediately fixed in 4% paraformaldehyde and then paraffin embedded. The presence of viable endometiosis was confirmed by conventional hematoxylin and eosin staining in one 5 µm thickness section per implant. The histologic diagnosis of endometriosis was based on the morphologic identification of endometrial glandular tissue and stroma. An individual other than the authors evaluated three sections from each implant in a blinded fashion.

Finally, the expression of Bcl-2 and VEGF were detected in serial sections by immunohistochemistry according to the laboratory protocol. Sections were deparaffinized in xylene and rehydrated through graded alcohols to distilled water. In sections used to detect VEGF this was followed by microwaving in 0.01 mol/L sodium citrate buffer for antigen retrieval. Incubation with 0.01% trypsin for 30 min at 37°C was used to detect Bcl-2, and steam-heat incubation with EDTA for 30 min at 97°C pH 8 was used for VEGF. Endogenous peroxidase was removed by treatment with 0.3% hydrogen peroxide for 30 min at 37°C, after which nonspecific binding was blocked by incubation with normal goat serum. Tissue sections were incubated with anti-human Bcl-2 rabbit polyclonal antibody (1:100, Santa Cruz Biotechnology, Santa Cruz, CA, USA) or VEGF anti-human rabbit monoclonal antibody (1:200, Zeta, Sierra Madre, CA, USA) overnight at 4°C, then incubated with anti-rabbit-peroxidase conjugate (Zhongshan Ltd., Beijing, China) for 60min at 37°C. Binding was visualized by incubating sections with 3, 3′-Diaminobenzidine (DAB). After rinsing, slides were counterstained with hematoxylin, dehydrated through a graded ethanol series to xylene, and then coverslips put in place. Negative control specimens were processed by omitting the primary antibody. The intensity of Bcl-2 and VEGF staining was assessed in a blind fashion at 200 magnifications by microscopy (Leica, Germany). Images were captured and the gray value was measured using Leica Qwin, standard V2.8 software. A quantitative assessment method was used to compare the gray value of 3 randomly selected fields of each tissue specimen.

### Measure of IL-8 and VEGF

At the end of the experimental period, animals were sacrificed, the blood and PF were collected, and PF and serum levels of VEGF and IL-8 were measured by standard cytokine-specific enzyme-linked immunosorbent assay (ELISA) using commercial kits (Adlitteram Diagnostic Laboratories, USA). All measurements were performed in duplicate, according to the manufacturer's instructions. The sensitivity of the kits was approximately 1 pg/ml for both VEGF and IL-8.

### Statistical analysis

All data are expressed as mean ±SD. Differences among groups were analyzed by one-way analysis of variance (ANOVA), and the post hoc Student-Newman-Keuls (SNK) method was used for multiple comparison. The P-value reported was two-sided and value of *P* < 0.05 was considered statistically significant. All analyses were performed using the SPSS software (Version 11.0, SPSS Inc., USA).

## RESULTS

The behavior of the mice during the experiment was normal. No evidence of toxicity was noted in both the sIL-1 RII group and IL-1 group. The body weights of three groups (20.1±1.9 g of sIL-1 RII group, 19.7±2.4 g of IL-1 group, and 20.3±2.2 g of control) were similar (*P* > 0.05).

**Fig.1:**
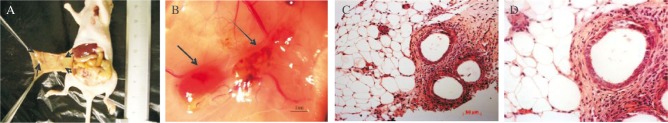
The lesions on day 14 after transplantation (A), The lesion viewed by stereo microscope, 12×(B). The morphology of the lesions at day 14 after transplantation, 200×(C) and 400×(D).

Of a total of 19 mice which received endometrial fragments, all mice developed 4 endometriotic lesions in the peritoneum at 2 weeks ***(Fig. 1 A)***. Macroscopically, the deposits appeared as reddish cystic areas and were found to be well vascularized and attached to the abdominal wall peritoneum ***(Fig. 1 B)***. Histological sections stained with hematoxylin and eosin detected the lesions taken from all groups at day 14 after transplantation revealed typical endometriotic lesions; the morphology of endometriotic lesions in all treatment groups was diverse, with typical endometrium glands and stroma as well as tubal metaplasia with large cysts and flattened epithelium. The observed morphology of the lesions was similar to that seen in human endometriosis ***(Fig. 1 C and 1D)***.

### Effects of sIL1-RII on the growth of endometriotic implants

**Fig.2:**
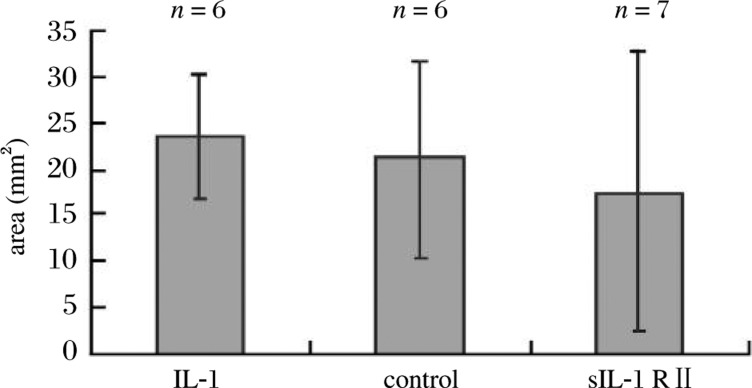
The average area of lesions per mouse in each of the three groups (sIL-1R II treated group, IL-1 treated group and control group treated with saline) at day 14 after transplantation (*P* = 0.645).

After two weeks of treatment, the average area of ectopic endometrial lesions in the sIL-1 RII treated nude mice decreased compared with the other two groups, but there was no significant difference among the three groups ***(Fig. 2)***.

### Treatment of sIL1-RII inhibits the expression of VEGF and Bcl-2 in endometriotic lesions

**Fig.3:**
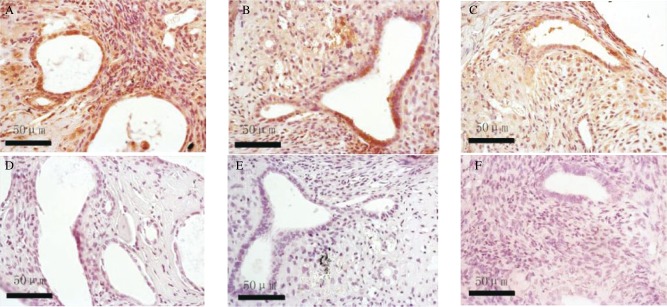
The expression of Bcl-2 on day 14 after transplantation in the 3 groups: the IL-1 treated group (A) and the negative control (D), the saline treated control group (B) and the negative control (E), and the sIL-1 RII treated group (C) and the negative control (F)

**Fig.4:**
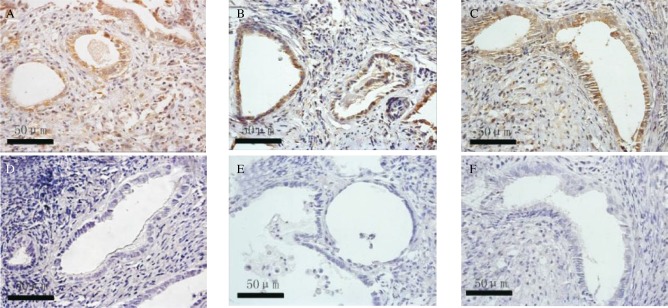
The expression of VEGF on day 14 after transplantation in the 3 groups: the IL-1 treated group (A) and the negative control (D), the saline treated control group (B) and the negative control (E), and the sIL-1 RII treated group (C) and the negative control (F).

**Fig.5:**
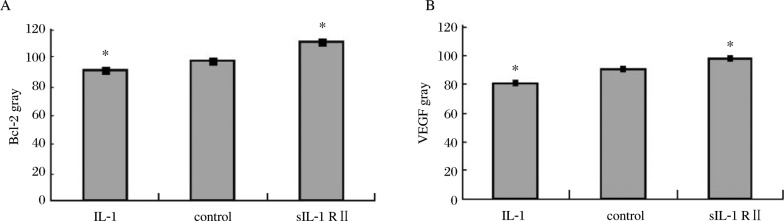
The gray values (inversely proportional to protein expression) of Bcl-2 (A) and VEGF (B) of the immunohistochemical slides of the 3 groups. Significantly different from the control (*P < 0.05).

The immunohistochemical staining of the slides were reviewed on three separate occasions by different combinations of investigators. The Bcl-2 and VEGF were mainly located in the plasma membrane and membranes of intracellular organelles. Expression of Bcl-2 was present in both epithelial and stromal cells of the endometrium ***(Fig. 3)***, while the expression of VEGF was mostly limited to the epithelium ***(Fig. 4)***. The negative control sections showed an absence of specific staining. ***Fig. 5*** showed the gray value of the lesions' glandular epithelium staining for each of the three groups. The expression of protein was the inverse of the gray value, so the expression of VEGF and Bcl-2 were highest in the IL-1 group, but lowest in the sIL-1 RII group and both proteins in each group were significantly different (*P* < 0.05) from the control group values.

### The IL-8 and VEGF levels in the peritoneal fluid and serum

**Fig.6:**
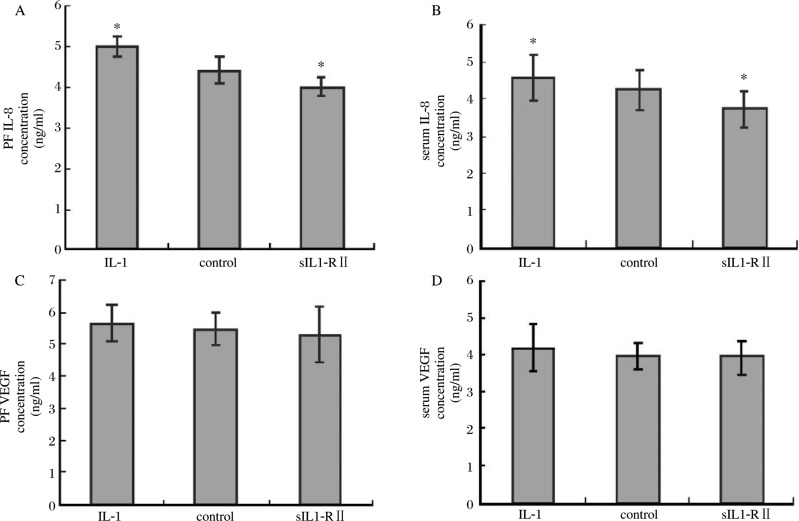
The IL-8 and VEGF concentrations in the PF and serum for each group of animals. Significantly different from control (*P < 0.05). A: The IL-8 concentration in PF; B: The IL-8 concentration in serum; C:The VEGF concentration in PF; D: The VEGF concentration in serum.

In the PF and serum, the concentrations of IL-8 and VEGF were lowest in the sIL1-RIIgroup and highest in the IL-1 group. The differences in IL-8 concentration between any two groups reached statistical significance (*P* < 0.05), but the VEGF did not (*P* > 0.05) ***(Fig. 6)***.

## DISCUSSION

It has been demonstrated that the expression of IL-1ß by peritoneal macrophages was elevated during the initial stages of endometriosis but the IL-1ra was usually elevated during the late stages of the disease[15]. An up-regulation of IL-1 expression was recently observed in the eutopic endometrial tissue of endometriosis patients[23]. It has also been found that sIL-1 RII binds and blocks processing of the IL-1ß precursor, inhibits its maturation, loses affinity for the IL-1ra and does not therefore interfere with IL-1ra mediated inhibition of IL-1 effects[24]. Finally, sIL-1 RII appears to act by sequestering IL-1ß within the extracellular compartment, thus restricting its availability and interaction with IL-1 RI.

IL-1 induces an angiogenic phenotype in endometriotic cells and stimulates the secretion of VEGF. Recently, several studies have proposed that angiogenesis represents an important step during the process of endometriosis, because similar to tumour metastases, endometriotic implants require neovascularization to establish, proliferate and invade[25]–[27]. The endometrium has angiogenic potential[28], and endometriotic lesions are larger in areas with a rich blood supply[29]. In a recent study about the growth of human menstrual endometrium in nude mice, a high VEGF score was observed in stromal cells as early as 5 days after transplantation, suggesting that an active vascular network is a necessary condition for the survival of the graft[30]. Our results suggest that sIL-1 RII treatment throughout the development of endometriosis decreases the expression of VEGF protein, which then affects the angiogenesis of the lesions. Although, the change of VEGF in PF and serum showed no significant difference (*P* > 0.05) between the three groups after two weeks of treatment of nude mice, this may be caused by the short treatment time.

In endometriosis, abnormal adhesion of endometrial fragments to ectopic sites could be due to an increase in the adhesive and proliferative properties of the menstrual endometrium, a decrease in apoptosis, and anomalities in the target tissue microenvironment. Previous studies demonstrated that endometrial cells (ECs) from women with endometriosis exhibited low apoptosis, when compared to ECs from women without endometriosis[31]. Dmowski and colleagues[32], using a cell death detection ELISA assay, demonstrated that endometrial apoptosis in the eutopic endometrium from women with endometriosis was lower than controls, and that endometrial apoptosis in the ectopic endometrium further decreased when compared to the eutopic endometrium. The evidence suggested that the normal balance between proliferative and apoptotic cyclicity of the endometrium was disturbed in women with endometriosis[33], with a reduced apoptosis which was observed primarily in the early proliferative and late secretory phases of the menstrual cycle[34]. The disturbed expression of IL-1 RII on the eutopic endometrium resulted in disturbances in normal macrophage trafficking into the eutopic endometrium in women with endometriosis. There may also be a positive correlation between apoptosis and macrophage numbers in the eutopic endometrium[35]. These results provided further evidence for an intrinsic abnormality in apoptotic sensitivity of the endometrium of women with endometriosis. The Bcl-2 is defined as a new class of proto-oncogenes that block cell death without promoting cell proliferation[36]. Under normal conditions, epithelial cells require interactions with the extracellular matrix to survive and grow. Apoptosis is thought to prevent the dissemination and attachment of differentiated epithelial cells at inappropriate locations when they lose contact with the matrix. It is likely that in healthy women, ECs and tissue fragments expelled during menses do not survive in ectopic locations because of programmed cell death. Disruption of cell matrix interactions induces cell death through a process called anoikis[37]. This might prevent detached normal cells from implanting in a new and inappropriate location. Endometriotic lesions show an increase in Bcl-2 expression compare with the endometrium and such an elevated Bcl-2 expression could promote the survival of endometriotic tissue at ectopic sites[38]. Our results demonstrate that in the formed lesions, sIL-1 RII decreased the expression of Bcl-2 and VEGF compared with both control and IL-1 groups, and may contribute to decreasing the size of already formed lesions in the endometriosis nude mouse model.

IL-8 is elevated in the PF of women with endometriosis[39]–[41], and the IL-8 level is also correlated with the severity of the disease[40]. It has recently been shown that IL-8 stimulates the adhesion of endometrial cells to fibronectin[42]. Thus, IL-8 may be relevant for stimulating the attachment of endometrial implants in the pathogenesis of endometriosis, and the adherence of ECs induces further IL-8 expression by an integrin-dependent mechanism[43].

Our previous results demonstrated that IL-8 secretion induced by IL-1β was significantly decreased after infection with recombinant adenovirus expressing IL-1RII[18]. Now, we have found that in an endometriosis nude mice model, the IL-8 concentration in the peritoneal fluid and serum of the sIL-1 RII treated group is significantly lower than the other two groups, and is in fact increased in the IL-1 treated group. Although the cytokine network in the endometrium is complex, our results provide further insights into the mechanism of immunologic modulation of sIL-1 RII during the process of endometriosis.

Taken together, these findings further support the contention that the pathogenesis of endometriosis is multifactorial and involves many mediators. Presently, endometriosis is mainly treated by estrogen antagonist, which only suppresses symptoms, but will not eradicate the ectopic implants. Moreover, there are significant side effects. Alternatively, endometriosis can be treated surgically, but symptoms recur in time in a majority of women. An effective therapeutic agent for endometriosis would be a compound that not only prevents the development of endometriotic lesions but is also effective against the growth of established lesions. Our results suggest that sIL-1 RII may provide a new clinical strategy for the treatment of endometriosis.
